# Urinary Calculi: A Microbiological and Biochemical Analysis at a Tertiary Care Hospital in Eastern Nepal

**DOI:** 10.1155/2020/8880403

**Published:** 2020-09-12

**Authors:** Pratima Shah, Ratna Baral, C. S. Agrawal, Madhab Lamsal, Dharanidhar Baral, Basudha Khanal

**Affiliations:** ^1^Department of Microbiology & Infectious Diseases, B.P. Koirala Institute of Health Sciences (BPKIHS), Dharan, Sunsari 56700, Nepal; ^2^Department of Surgery, B.P. Koirala Institute of Health Sciences (BPKIHS), Dharan, Sunsari 56700, Nepal; ^3^Department of Biochemistry, B.P. Koirala Institute of Health Sciences (BPKIHS), Dharan, Sunsari 56700, Nepal; ^4^School of Public health and Community Medicine, B.P. Koirala Institute of Health Sciences (BPKIHS), Dharan, Sunsari 56700, Nepal

## Abstract

**Background:**

The occurrence of urinary tract infection in presence of urolithiasis is frequently noted; however, microbial agents of urolithiasis and their antimicrobial susceptibility patterns remain underinvestigated. This study aimed to identify the microorganisms isolated from urine and stone matrices to determine their antimicrobial susceptibility, to find the association between the pathogens of urine and stone matrices, and to perform the biochemical analysis of stones.

**Methods:**

A total of 88 cases of urolithiasis admitted for elective stone removal at Department of surgery, B.P. Koirala Institute of Health Sciences (BPKIHS), were enrolled. Preoperative urine culture and postoperative stone culture were performed. Isolation, identification, and AST were done by the standard microbiological technique. Further qualitative biochemical analysis of stones was also attempted.

**Result:**

Among 88 stone formers recruited, culture of urine, whole stone, and nidus yielded the growth of bacteria 44, 32, and 30, respectively. Bacteria isolated from urine culture correlated with those from stone matrices with a sensitivity of 90%, specificity of 79.69%, PPV of 63.64%, and NPV of 95.45%. *Escherichia coli* (46.7%) was the most common bacteria followed by *Klebsiella pneumoniae* (16.7%) and *Proteus mirabilis* (13.3%) from urine and stone cultures. Almost all the uropathogens isolated were susceptible to commonly used antibiotics. Calcium oxalate (84.1%) was common biochemical constituent found in stone formers followed by calcium oxalate + phosphate (8%).

**Conclusions:**

The association of microorganism isolated from urine and nidus culture was significant that can predict the source of infective stone; however, in some cases, microorganisms and the antimicrobial susceptibility pattern from urine and nidus were different. This study emphasizes the use of appropriate antimicrobial agents to prevent the regrowth of residual stones and minimize the risk of infectious complications after surgical removal of stones.

## 1. Introduction

Urolithiasis is one of the frequently encountered urological disorders, common throughout the world, and is highly prevalent in Nepal [1].

The association between urolithiasis and urinary tract infections (UTIs) is well known and is frequently detected. Their interrelationship can be defined in two ways: urolithiasis following UTIs, i.e., “infection-induced stones” or urinary stone with subsequent UTIs as its complications [2]. Although the calculi themselves are the important source of secondary infection, their prevalence, causative microorganisms, and antimicrobial susceptibility patterns in Nepal remain underinvestigated. Approximately, 15% of urinary stones are infective stones. However, formation of all noninfective urinary calculi is a consequence of unknown changes in kidney tissue or metabolic disturbances [3].

The history describes infective stone or struvite as the most common type of urinary stones containing magnesium ammonium phosphate, whereas urea-splitting bacteria like *Proteus* spp., *Staphylococcus aureus*, *Klebsiella* spp., *Providencia* spp., and *Ureaplasma urealyticum* are commonly responsible for struvite stone. The antimicrobial agents could not invade, where these bacteria lie within the interspace of stones in urinary tract. Thus, the outcome is progressive expansion of stones because of persistent infection over a period of weeks or months [2, 4].

To prevent infectious complications and subsequent recurrence of residual stones after surgical removal, association of microbes in the stone and proper antibiotic therapy are essential. The selection of antibiotic agents is based on bacteria isolated from urine culture; however, the efficacy of treatment of stone bacteria cannot be ascertained due to uncertainty in similarity of stone and urine bacteria.

Similarly, biochemical profile of the patient should be evaluated to ameliorate metabolic disorder and to inhibit reoccurrence of metabolic stone. Therefore, characterization of calculus material aids knowledge to establish the management of a patient postoperatively [5, 6].

Thus, this study was aimed to identify the type of bacteriology of urine and stone matrices, determine their antimicrobial sensitivity pattern, and find the concordance between urine and stone bacteria. It was also aimed to determine the biochemical composition of urinary calculi to know the origin and etiology of urinary stones.

## 2. Methods

This cross-sectional descriptive study was conducted in the Department of Microbiology in collaboration with Department of General Surgery and Biochemistry. A total of 88 patients having urolithiasis, admitted at B. P. Koirala Institute of Health sciences, Dharan, Nepal, for elective stone removal between July 2014 and June 2015 were enrolled. Informed consents were taken from the patients, and ethical clearance was obtained from the Institutional Review Committee of BPKIHS (Code No: IERB/277/014).

The demographic details and associated factors with urolithiasis such as past and family history of stone disease, history of hypertension, diabetes mellitus, and gout were recorded in predesigned proforma.

### 2.1. Sample Preparation, Isolation, and Identification of Bacteria [7–9]

The midstream urine specimen was cultured from each patient before surgical stone removal. The stone was also collected from the same patient after surgery.

Using the semiquantitative method, 10^5^ colony-forming units per milliliter (CFU/ml) of urine was considered as significant bacteriuria and further processed for identification of organisms. The bacterial pathogens were identified up to species level by standard microbiological techniques like colony morphology, Gram staining, and several biochemical tests.

Antimicrobial susceptibility of the isolates was determined by the Kirby–Bauer disc-diffusion method on Muller–Hinton agar (MHA) according to Clinical Laboratory Standards Institute (CLSI) guidelines [10].

### 2.2. Preparation and Processing of Urinary Stone [2]

After the surgical removal, the stone sample was washed several times with sterile water and each stone was then divided into two parts, as symmetrical as possible. For the first part, stone matrices were taken from “nidus” (nucleus) portion by scraping. For the second part, the stone was crushed into powder by sterilized mortar and pestle and was then used for bacterial culture and chemical analysis of the “whole stone”. All samples derived from two locales of individual stones (including nidus and whole stone) were then inoculated in brain-heart infusion (BHI) broth, incubated at 35°C for 6–8 hours, inoculated onto blood, MacConkey, CLED agar (HiMedia Laboratories), and incubated at 35°C for 24 and 48 hours, respectively.

Bacteria isolated thus were identified by standard biochemical tests. Antimicrobial susceptibility was determined by the disc diffusion assay on MHA (HiMedia Laboratories) following the standards of CLSI [10].

### 2.3. Analysis of Chemical Compositions of Stones

The qualitative biochemical analysis of compositions of each stone was done for carbonate, calcium, magnesium, phosphate, oxalate, uric acid, and cysteine, using stone powder derived from the second part of stone sections (as aforementioned) that was left after bacterial culture [11].

### 2.4. Statistical Analysis

All data collected were entered in MS excel 2007 and analysed using SPSS 21.0. For descriptive analysis, percentage and ratio were calculated with tabular and graphical presentation of analysis.

For inferential statistics, the chi-square test was applied to find out the relationship between dependent and independent variables. *P* values <0.05 were considered statistically significant.

## 3. Results

Out of eighty-eight patients, 50 were males with an average age of 38.3 (range: 17–67) years and 38 were females with an average age of 41.2 (range: 18–69) years. The male: female ratio was 1.3 : 1. Forty had nephrolithiasis (45.5%), thirty-seven had ureterolithiasis (42%), and eleven had urinary bladder stone (12.5%).

The comorbidities were observed in 23 (27.1%) patients. Hypertension was present in 18 (78.3%) patients, and 6 (26.1%) had diabetes mellitus. Only 1 (4.35%) of the patients was found to have gout, and a past history of urolithiasis was present in 2 (2.272%).

### 3.1. Urine and Stone Matrices Culture

Bacterial growth was obtained in urine of 44 patients (50%). The most common organism isolated was *Escherichia coli* (20 (45.5% of total isolates)) followed by *Klebsiella pneumoniae* (7 (15.9%)), *Proteus mirabilis* (4 (9.1%)), *Enterococcus faecalis* (4 (9.1%)), and *Staphylococcus aureus* (3 (6.8%)).

When attempted stone culture, whole stone of 32 (36.4%) out of 88 patients yielded the growth of bacteria, whereas nidus culture bacteria in 30 (34.1%). The most common organism isolated from whole stone and nidus culture was *Escherichia coli* followed by *Klebsiella pneumoniae*, *Proteus mirabilis*, *Enterococcus faecalis*, and *Staphylococcus aureus*. Infective stone was found in 34.1% of the cases. The culture results and bacterial isolates are shown in Table 1.

### 3.2. Association of Bacteria Isolated from Urine and Stone Matrices Culture

Bacteria isolated from urine were similar to those obtained from stone matrices. Considering culture of stone matrices a gold standard, sensitivity and specificity of urine culture were found to be 93.3% and 72.4%, respectively. The PPV and NPV were 63.6% and 95.5%, respectively (Table 2).

### 3.3. Similarity of Bacteria Isolated from Urine and Stone Matrices Culture

#### 3.3.1. Concordance between Microorganisms Isolated from Urine and Stones Matrices Culture

Some bacteria isolated from urine were phenotypically similar to those from stone matrices. Out of 30 stone formers, a total of 30 bacteria were isolated. Among those 30 stone formers, 28 had bacterial isolates in both urine and stone matrices, whereas no organism was obtained from urine in only 2 of the patients. Among 28 stone formers, 24 had the same organism and 3 had different strains of *E*. *coli* found in urine and stone matrices, whereas the remaining 1 had different isolates, i.e., *E*. *coli* from urine and *K*. *oxytoca* from stone matrices. The bacteria isolated from urine were common to stone matrices in context of whole stone culture also. The culture results are shown in Tables 3 and 4.

### 3.4. Antimicrobial Susceptibility Pattern (AST) of Urinary Pathogen

#### 3.4.1. Antimicrobial Susceptibility Pattern of Gram-Negative Bacteria

**Figure 1:**
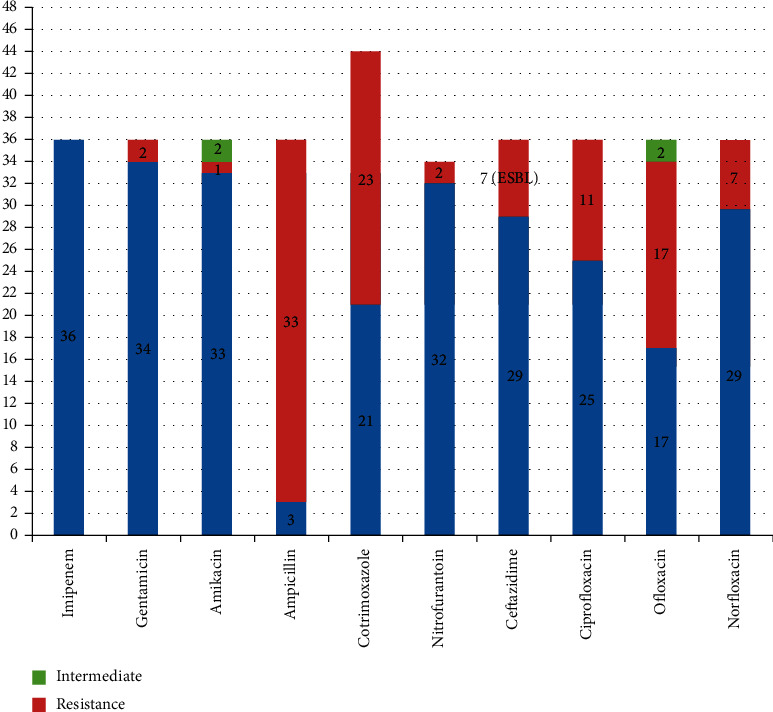
Antimicrobial susceptibility pattern of Gram-negative bacteria.

All Gram-negative bacteria isolated were sensitive to imipenem (100%) followed by gentamicin (94%), amikacin (91%), nitrofurantoin (88%), ceftazidime (87.9%), and cotrimoxazole (58%), and most of them were resistant to ampicillin (91.6%) and ofloxacin (69%). Out of 20 *E*. *coli*, seven (35%) *E*. *coli* were ESBL producers (Figure 1).

### 3.5. Antimicrobial Susceptibility Pattern of Gram-Positive Bacteria

All Gram-positive bacteria, *S*. *aureus*, *E*. *faecalis,* and CONS isolated, were sensitive to vancomycin (100%), linezolid (100%), gentamicin (100%), and nitrofurantoin (100%) followed by amikacin (87.5%), norfloxacin (87.5%), ciprofloxacin (75%), cotrimoxazole (62.5%), and ofloxacin (50%). Out of 3 isolates of *S*. *aureus*, 2 were MRSA.

### 3.6. Antimicrobial Susceptibility Pattern of Bacteria Isolated from Stone Matrices (Nidus)

#### 3.6.1. Antimicrobial Susceptibility Pattern of Gram-Negative Bacteria

**Figure 2:**
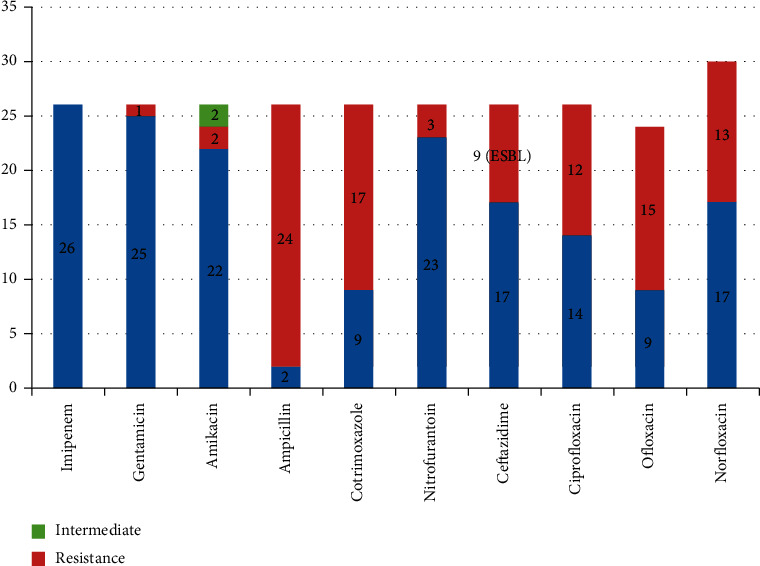
Nidus-antimicrobial susceptibility pattern of Gram-negative bacteria.

All Gram-negative bacteria isolated were sensitive to imipenem (100%) followed by gentamicin (96%), amikacin (84.6%), nitrofurantoin (88.5%), ceftazidime (65.4%), norfloxacin (65.4%), ciprofloxacin (53.8%), cotrimoxazole (34.6%), and ofloxacin (34.6%) and were resistant to ampicillin (92%). Out of total isolates, 9 were ESBL producers (Figure 2).

### 3.7. Antimicrobial Susceptibility Pattern of Gram-Positive Bacteria

All Gram-positive bacteria were sensitive to vancomycin (100%), linezolid (100%), gentamicin (100%), and nitrofurantoin (100%) followed by ceftriaxone (50%), cotrimoxazole (50%), amikacin (50%), and ciprofloxacin (50%), and most of them were resistant to ofloxacin (100%). Out of 3 isolates of *S*. *aureus*, 2 were MRSA.

### 3.8. Stone Analysis

The qualitative biochemical stone analysis was attempted in all the stones, and the most common type was found to be calcium oxalate (74 (84.1%)) followed by calcium oxalate phosphate (7 (8.0%)), ammonia (3 (3.4%)), calcium uric acid (2 (2.3%)), and calcium carbonate (1 (1.1%)).

### 3.9. Bacteria Associated with Stone

**Figure 3:**
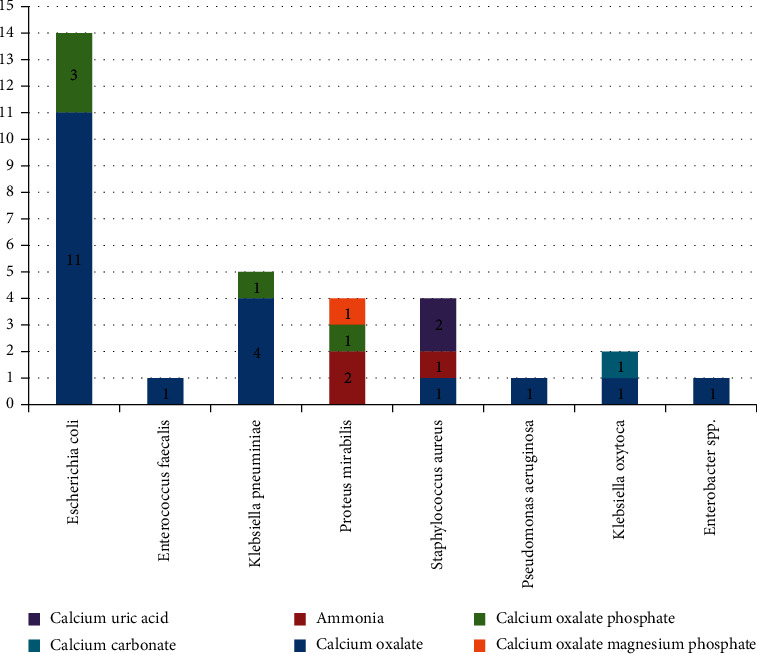
Stone bacteria with stone composition.

Among 74 calcium oxalate stones, 23 (71.9%) had association with stone matrices (nidus) bacteria. Most common stone formers were *E*. *coli* followed by *K*. *pneumoniae*. *Proteus mirabilis* was associated with ammonia and calcium oxalate with magnesium and phosphate. Bacterial isolates from nidus culture were associated with almost all types of urinary stones (Figure 3).

## 4. Discussion

This study made an attempt to identify the type of bacteriology of urine and stone matrices, to determine their antimicrobial sensitivity pattern and to find the concordance between urine and stone bacteria in patients with urolithiasis.

### 4.1. Demographic Factors

The age of the patients in our study ranged from 17 years to 69 years, frequency being highest in age groups of 30–39 years (29.5%), a finding contradictory to previous studies conducted which documented that the frequency of urinary stones increased with age.

Among the study participants, 50 (56.8%) were male and 38 (43.2%) were female. Although nephrolithiasis continues to be more common in men, the male-to-female ratio with urinary tract stones has narrowed from 3.1 to 1.3 from 1970 to 2000 [12] and from 1.6 : 1 to 1.2 : 1 from 1998 to 2003 [13]. The striking new trends of increased incidence of stone formation in women might be due to associated risk factors such as increasing obesity, dietary changes, and change in fluid intake patterns. In the context of our study, male preponderance was observed. It can be attributed to effect of sex hormones on some lithogenic risk factors and concentration of lithogenic factors in the urine which is greater in men than that in women [14–17].

By occupation, 32 (36.4%) were housewives, 21 (23.9%) were involved in business, 13 (14.8%) in service, 9 (10.2%) in farming, 7 (8.0%) were students, and 6 (6.8%) were laborers. As reported by Vhlensieck et al. [18] and Kadir et al. [19], our data also showed that the frequent occupational group was prone to develop urinary stones with sedentary life styles like housewives, business (shopkeeper), and service holders. Sedentary lifestyle predisposes to sedimentation of urine, and crystals may be trapped by gravity in upward draining collecting tubules or in the inferior calices of the kidney [20].

Regarding comorbid conditions, 18 (20.5%) respondents were hypertensives and 6 (6.8%) were diabetics. Hypertension with diabetes was seen in 2 cases, while 1 had gout (1.1%). Several studies have established hypertension as an independent risk factor of urolithiasis [21–23] with a proposal that abnormalities in renal calcium metabolism exist among patients with hypertension, leading to increased urinary calcium excretion [24]. Our findings of 12 hypertensive patients having calcium oxalate stones and one with calcium uric acid stone are in concordance with the abovementioned studies.

Urinary stone disease has been increased globally and occurred more frequently in subjects with diabetes than nondiabetics with a predominance of urolithiasis with uric acid [25–27]. The reason for a higher occurrence of urolithiasis in diabetes mellitus has been explained as insulin resistance and lower urine pH through impaired kidney ammonia genesis, promoting uric acid stone formation [28]. Although a low urinary pH plays a major role in the formation of uric acid kidney stones, a defect in renal acid excretion also could lead to hypocitraturia, an important risk factor for calcium stones [29]. In our study, the patient with diabetes had infective stones. The chemical composition of stones varied in different patients such as calcium oxalate, ammonia, and calcium oxalate phosphate, which showed different results than the study done by Medyen et al. and Chu et al. [25, 27].

In the study by Alvarez-Nemegyi et al., 39% of patients with primary gout had urinary stones, of which about 30% were silent and diagnosed only by ultrasonography, meaning the prevalence of urolithiasis in gout is likely to be higher than commonly reported [30]. We could not comment upon the association of gout with urolithiasis in our study as we had only one such case. Perhaps, most of the cases with urolithiasis in gout remain silent as stated by Alvarez-Nemegyi et al. and thus are underreported.

Regarding stone locations, in the present study, 40 (45.5%) had nephrolithiasis, 37 (42.0%) ureterolithiasis, and 15 (12.5%) had urinary bladder stones. Upper urinary tract stone constituted 87.5% and lower urinary tract stone 12.5% in the ratio of 7 : 1. Our results have been consistent with study of Ahmed et al. [31] who reported increased frequency of renal stones. Kidney acts as a first barrier filter for crystals, thereby damaging tubular epithelium, which acts as a nidus for the stone formation. However, our result was different from the study of Venkatramana who observed the increased frequency of ureteric stone [32]. This variation might be due to selection of the patients irrespective of site of the stones.

Past history of urolithiasis was present in 2 (2.3%) cases. The reason for such low incidence of recurrence in our study may be because of the relatively shorter duration (1 year) of study, whereas many previous studies had followed up the patients for 10 years.

### 4.2. Urinary Tract Infection and Infective Stone in Urolithiasis

In this study, bacterial growth was obtained in urine of 50% of patients. Organisms isolated in decreasing order of frequency were *Escherichia coli* (20 (45.5%)), *Klebsiella pneumoniae* (7 (15.9%)), *Proteus mirabilis* (4 (9.1%)), *Enterococcus faecalis* (4 (9.1%)), and *Staphylococcus aureus* (3 (6.8%)).

Culture positivity of 50% in our study is less as compared to other studies [33, 34]. Our setting being a tertiary care hospital in a country where antibiotics are available over the counter could be considered contributing to less culture positivity. The patients might have reported to other health care settings and received antimicrobials before presenting to our hospital.

#### 4.2.1. Stone Matrices Culture and Infective Stone

Whole stone of 32 patients (36.4%) yielded the growth of bacteria, whereas nidus had a positive result in 30 (34.1%) of the cases. The common organisms isolated from whole stone and nidus were *Escherichia coli* followed by *Klebsiella pneumoniae*, *P*. *mirabilis*, *E*. *faecalis,* and *S*. *aureus.* Infective stone was found in 34.1%. The urease producing and citrate utilizing organisms formed 14 (46. 7%) and 12 (40%), respectively. Whole stone culture was positive in 2 patients with the same organism isolated from urine culture and sterile nidus culture, indicating the stone formation as infection induced.

It has been hypothesized that alteration in urinary enzymes, i.e., decreased urokinase and increased sialidase in urine, leads to the formation of mineralizable matrix. Microorganisms like *Proteus mirabilis* and *Escherichia coli* associated with infection-induced stones inhibited the urokinase and stimulated the sialidase activity leading to matrix formation, in turn causing increased crystal adherence to the renal epithelium [35].

An alternative explanation for the presence of bacteria within stone and urine is that of secondary ascending infection from the bladder urine. Penetration of bacteria in the stone prevents complete eradication of urinary tract infection by conventional antibiotic therapy, allowing the development of resistant organisms with intermittent shedding in urine. It is a vicious cycle of infection bringing about stone formation and stone formation causing infection [36, 37].

#### 4.2.2. Association of Bacteria Isolated from Urine and Stone Matrices Culture of the Patients

A total of 30 bacteria were isolated from stone formers and 28 had isolates in both urine and stone matrices, whereas in 2 patients, urine was sterile. Among 28 stone formers, in both urine and stone matrices, 24 had the same organism phenotypically and 3 had different strains of *E*. *coli* found in urine and stone matrices, while the remaining one had different isolates, i.e., *E*. *coli* from urine and *K*. *oxytoca* from stone matrices. Bacteria isolated from urine were common to stone matrices in context of whole stone culture also. The concordance rate between urine culture and stone culture varied from 16% [38], 48 4% [39], 50% [40], and 70% [4].

Our result also showed that the bacteria isolated from urine were associated significantly (*P* value < 0.001) to nidus of stone which is considered gold standard. Though the concordance rate and sensitivity were high (93.3%), the specificity and PPV were only 72.4% and 63.6%, respectively. Antimicrobial susceptibility pattern of the bacteria isolated from infective stone was different than that from urinary isolates. Most of the microorganisms isolated from infective stone were multidrug resistant.

### 4.3. Stone Analysis

Stones from all 88 patients were subjected to qualitative biochemical analysis. The most common type of stone was calcium oxalate (*n* = 74, 84.1%); others were calcium oxalate phosphate (*n* = 7, 8.0%), ammonia (*n* = 3, 3.4%), calcium uric acid (*n* = 2, 2.3%), and calcium carbonate (*n* = 1, 1.1%).

The infective stone accounted for 23 (71.9%) out of the 74 calcium oxalate stones determined. The most common stone formers were *E*. *coli* followed by *K*. *pneumoniae*. *Proteus mirabilis* was associated with ammonia and calcium oxalate with magnesium and phosphate. *S*. *aureus* formed the calcium oxalate stone in mixed form with uric acid and ammonia. The bacterial isolates from nidus culture were associated with almost all types of urinary stones.

Several studies have found out the composition of staghorn stones which were considered to be a major portion of infective stones. Earlier studies demonstrated that calcium phosphate and struvite were major constituents of 75% of infective stones [41].

There was only one struvite stone in our study that too occurred in combination with calcium oxalate. This could be attributed to the smaller number of urease producing bacteria *Proteus* spp. In addition, other factors such as anatomic or functional abnormalities of urinary tract and diet may be responsible.

The present study supports studies from north India and Brazil, which reported calcium oxalate as the most common constituent of infective stones [42, 43].

As reported in literature, dietary habits are associated with urinary tract stones. Increased animal protein, high calorie content, as well as calcium and oxalate intake in the diet [44] are considered nutritional risk factors for urolithiasis. A study from Marathwada, India, has shown that the incidence of urolithiasis increases with consumption of diet containing groundnuts, tomato, spinach, and animal proteins and with a greater use of salt. Stone composition, urinary risk factors, and dietary analysis suggest that diet, dehydration, and poor nutrition are the causative factors of stone diseases [45].

Our study showed that calcium oxalate was the most common urinary tract stones, a finding consistent with that of Trinchieri [46] and Pandeya et al. [1]. One of the reasons for the occurrence of this type of stone has been cited as the consumption of nonvegetarian diet by the subjects as animal protein is found to lower the citrate excretion and increase calcium and uric acid excretion.

Diet with high oxalate content and high carbohydrate intake is also thought to be responsible for the occurrence of oxalate stones, particularly rice, and is found to increase the acidity of urine favoring calcium oxalate stone formation [1].

The results of the present study have indicated that infective stones constitute an accountable portion of urolithiasis with several types of microorganisms and with variable degrees of antimicrobial resistance. To understand the recurrent urinary infections, it is necessary to identify organism found deep in the stone matrices, which is not easily accessible to antimicrobial agents. Thus, this study has tried to aid information regarding the role of microbial agents as etiology of stone formation along with their antimicrobial susceptibility pattern so that appropriate antimicrobial therapy can be instituted not only for eradication of these agents but also for the prevention of recurrence.

## 5. Conclusion

The present study underscores the importance of microbiological analysis of stones for complete sterilization of urinary system and prevention of recurrence. In addition, this study recommends a further work on microbiological analysis of stones along with the correlation of various metabolic and dietary factors which may provide better insights regarding the origin of the stones in the urinary tract.

## Figures and Tables

**Table 1:** Pattern of bacteria isolated from urine and stone matrices culture.

Bacteria	Urine	Whole stone	Nidus
	No. of patients (%)	No. of patients (%)	No. of patients (%)
*Escherichia coli*	20 (45.5)	14 (43.8)	14 (46.7)
*Enterococcus faecalis*	4 (9.1)	1 (3.1)	1 (3.3)
*Klebsiella pneumoniae*	7 (15.9)	5 (15.6)	5 (16.7)
*Proteus mirabilis*	4 (9.1)	4 (12.5)	4 (13.3)
*Staphylococcus aureus*	3 (6.8)	4 (12.5)	3 (10.0)
*Citrobacter koseri*	2 (4.5)	0	0
*Pseudomonas aeruginosa*	1 (2.3)	1 (3.1)	1 (3.3)
CONS	1 (2.3)	0	0
*Klebsiella oxytoca*	2 (4.5)	2 (6.3)	1 (3.3)
*Enterobacter spp*.	0	1 (3.1)	1 (3.3)
Total	**44 (100)**	**32 (100)**	**30 (100)**

**Table 2:** Association of bacteria isolated from urine and stone matrices culture.

Test	Test status	Urine culture		*P* value	Remarks
		Positive	Negative		
Nidus culture	Positive	28	2	<0.001	Significant
	Negative	16	42		
Total	44	44			

**Table 3:** Similarity in urine and stone matrices (nidus) culture.

Nidus bacteria	Urine bacteria							Total
	*E*. *coli*	*E*. *faecalis*	*K*. *pneumoniae*	*P*. *mirabilis*	*S*. *aureus*	*P*. *aeruginosa*	*K*. *oxytoca*	
*E*. *coli*	14	0	0	0	0	0	0	14
*E*. *faecalis*	0	1	0	0	0	0	0	1
*K*. *pneumoniae*	0	0	5	0	0	0	0	5
*Proteus mirabilis*	0	0	0	4	0	0	0	4
*S*. *aureus*	0	0	0	0	2	0	0	2
*P*. *aeruginosa*	0	0	0	0	0	1		1
*K*. *oxytoca*	1	0	0	0	0	0	0	1
Total	15	1	5	4	2	1	1	28

**Table 4:** Similarity in urine and stone matrices (whole) culture.

Whole stone bacteria	Urine bacteria							Total
	*E*. *coli*	*E*. *faecalis*	*K*. *pneumoniae*	*P*. *mirabilis*	*S*. *aureus*	*P*. *aeruginosa*	*K*. *oxytoca*	
*E*. *Coli*	14	0	0	0	0	0	0	14
*E*. *faecalis*	0	1	0	0	0	0	0	1
*K*. *pneumoniae*	0	0	5	0	0	0	0	5
*Proteus mirabilis*	0	0	0	4	0	0	0	4
*S*. *aureus*	0	0	0	0	3	0	0	3
*P*. *aeruginosa*	0	0	0	0	0	1		1
*K*. *oxytoca*	1	0	0	0	0	0	1	2
Total	15	1	5	4	3	1	1	30

## Data Availability

The data used to support the findings of this study are available from the corresponding author upon request.

## Abbreviations

AST: Antimicrobial susceptibilitytest
BHI: Brain-heart infusion broth
CFU/ml: Colony-forming units per milliliter
CLSI: Clinical Laboratory Standards Institute
CLED: Cystein lactose electrolyte deficient
CONS: Coagulase negative staphylococci
ESBL: Extended spectrum beta lactamase
MHA: Muller–Hinton agar
MRSA: Methicillin-resistant *Staphylococcus aureus*
NPV: Negative predictive value
PPV: Positive predictive value
UTIs: Urinary tract infections
